# UNC-116 and UNC-16 function with the NEKL-3 kinase to promote axon targeting

**DOI:** 10.1242/dev.201654

**Published:** 2023-09-27

**Authors:** Cody J. Drozd, Christopher C. Quinn

**Affiliations:** Department of Biological Sciences, University of Wisconsin-Milwaukee, Milwaukee, WI 53201, USA

**Keywords:** Axon guidance, Axon transport, *C. elegans*, Kinesin, Neuronal development, Neuroscience

## Abstract

KIF5C is a kinesin-1 heavy chain that has been associated with neurodevelopmental disorders. Although the roles of kinesin-1 in axon transport are well known, little is known about how it regulates axon targeting. We report that UNC-116/KIF5C functions with the NEKL-3/NEK6/7 kinase to promote axon targeting in *Caenorhabditis elegans*. Loss of UNC-116 causes the axon to overshoot its target and UNC-116 gain-of-function causes premature axon termination. We find that loss of the UNC-16/JIP3 kinesin-1 cargo adaptor disrupts axon termination, but loss of kinesin-1 light chain function does not affect axon termination. Genetic analysis indicates that UNC-16 functions with the NEKL-3 kinase to promote axon termination. Consistent with this observation, imaging experiments indicate that loss of UNC-16 and UNC-116 disrupt localization of NEKL-3 in the axon. Moreover, genetic interactions suggest that NEKL-3 promotes axon termination by functioning with RPM-1, a ubiquitin ligase that regulates microtubule stability in the growth cone. These observations support a model where UNC-116 functions with UNC-16 to promote localization of NEKL-3 in the axon. NEKL-3, in turn, functions with the RPM-1 ubiquitin ligase to promote axon termination.

## Teaser

**Summary:** The kinesin-1 heavy chain promotes the localization and motility of the NEKL-3 kinase in axons. This process is required for axon targeting and its disruption may cause neurodevelopmental disorders.

## INTRODUCTION

Neurodevelopmental disorders (NDDs) such as autism, bipolar disorder, attention deficit hyperactivity disorder (ADHD) and epilepsy are thought to result from altered brain development. In recent years, genome sequencing has been used extensively to identify genes that are associated with these disorders. In most cases, this has been accomplished by identifying genes that are enriched for *de novo* mutations in affected populations (Awadalla et al., 2010; Deciphering Developmental Disorders, 2017; Iossifov et al., 2014; Kaplanis et al., 2020). These studies have resulted in the identification of hundreds of NDD genes. However, in most cases the roles of these genes in neuronal development are not well understood.

Defects in axon connectivity are thought to be a major underlying cause for NDDs. For example, functional MRI studies indicate that connectivity between brain regions is altered in individuals with autism relative to controls (Just et al., 2007, 2004; Schipul et al., 2011). Moreover, diffusion tensor imaging studies have found that specific long-range axonal tracts, such as the inferior longitudinal fasciculus, are altered in individuals with autism (Farías et al., 2017; Koldewyn et al., 2014; Lazar et al., 2014; Travers et al., 2012; Wolff et al., 2012). These observations suggest that disruptions in axonal connectivity contribute to NDDs. We are beginning to gain an understanding of how some of the NDD genes affect neuronal development. However, many studies of NDD genes have focused on dendrites and synapses. Much less is known about how loss of NDD gene function disrupts axon targeting.

Here, we focus on *unc-116*, the *Caenorhabditis elegans* ortholog of the *KIF5C* kinesin-1 heavy chain gene that has been implicated in NDDs including intellectual disability, epilepsy and autism (Awadalla et al., 2010; Cavallin et al., 2016; Coe et al., 2014; Jamuar et al., 2014; Kaplanis et al., 2020; Michels et al., 2017; Poirier et al., 2013; Wang et al., 2020; Willemsen et al., 2014). In particular, recurrent *de novo* variants at the E237 residue of the KIF5C protein have been associated with a syndrome characterized by intellectual disability, autistic traits, epilepsy, frontal cortical dysplasia and dysmorphic facial features (Cavallin et al., 2016; Jamuar et al., 2014; Michels et al., 2017; Poirier et al., 2013; Willemsen et al., 2014). In addition to the identification of this specific KIF5C syndrome, a role for other KIF5C variants in autism and intellectual disability has been supported by analysis of large-scale sequencing studies, revealing a statistically significant enrichment of KIF5C *de novo* variants in people with NDDs (Coe et al., 2014; Fu et al., 2022; Kaplanis et al., 2020; Wang et al., 2020).

The *KIF5C* gene encodes one of three human kinesin-1 heavy chain proteins (KHCs). In neurons, KIF5C can function with the kinesin-1 light chains (KLCs) to transport vesicles and organelles distally along the axon. For example, in mammalian neurons, the KHC uses the KLCs as adaptors to recruit lysosomes and transport them distally along axons (Farías et al., 2017). Likewise, in *C. elegans* neurons, the KHC functions with the KLCs to transport mitochondria distally along the axon (Sure et al., 2018). Moreover, the KHC functions with the KLCs to transport many different types of vesicles along the axon (Deng et al., 2014; Granger et al., 2014; Hoerndli et al., 2013; Saez et al., 2020; Schmidt et al., 2009). The KIF5C KHC can also function independently of the KLCs. For example, the KIF5C heavy chain can transport RNA granules in axons in the absence of KLCs (Kanai et al., 2004). Moreover, a process known as microtubule sliding occurs when the C-terminal domain of the KHC binds directly to microtubules and transports them as cargo along other microtubules (Lu et al., 2013; Winding et al., 2016). Despite our knowledge of how kinesin-1 promotes axon transport, little is known about how kinesin-1 promotes axon targeting.

Here, we uncover a novel mechanism whereby the UNC-116 KHC promotes axon termination by functioning with the UNC-16 (JIP3; also known as MAPK8IP3) cargo adaptor to interact with two regulators of microtubule stability, the kinase NEKL-3 and the E3 ubiquitin ligase RPM-1. We show that UNC-116, UNC-16 and NEKL-3 are all required for axon termination and that UNC-116 and UNC-16 are required for the localization and motility of NEKL-3 in the axon. Moreover, we also find that NEKL-3 functions with RPM-1 to promote axon termination. We also tested two mutations in UNC-116 that are equivalent to NDD-causing *de novo* mutations in KIF5C and find that both cause axon defects and disrupt NEKL-3 localization and motility in the axon. Taken together, these observations reveal a novel mechanism for the control of axon targeting and suggest that disruption of this process can lead to NDDs.

## RESULTS

### UNC-116 promotes axon termination

**Fig. 1.**
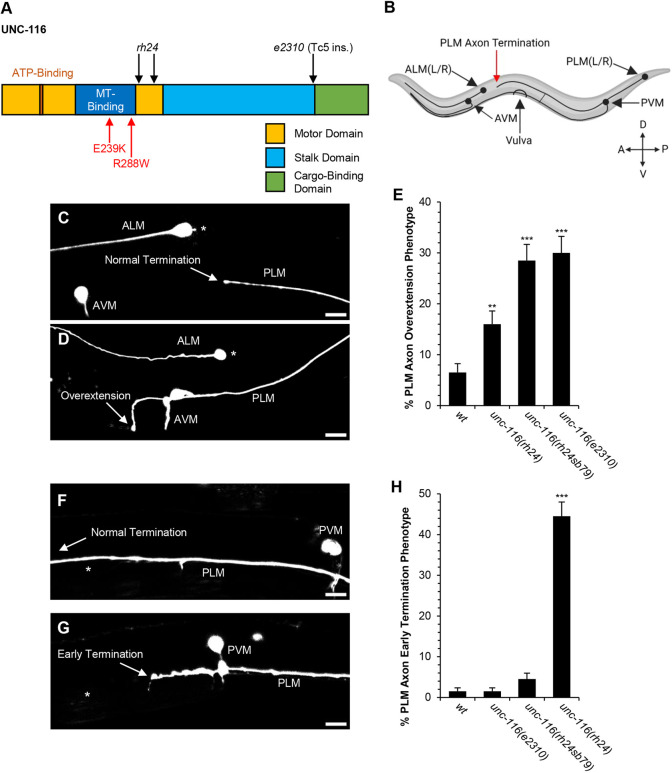
**UNC-116 loss of function causes axon overextension and UNC-116 gain of function causes early termination.** (A) The UNC-116 kinesin-1 heavy chain contains a motor domain, a stalk domain and a cargo-binding domain. Within the motor domain, there is an ATP-binding region and a microtubule-binding region. The *rh24* allele consists of two missense mutations within the motor domain (I304M+E338K, indicated by black arrows). The *unc-116* loss-of-function allele, *rh24sb79*, contains the *rh24* mutations as well as an additional missense mutation within the motor domain (not pictured). The E239K and R288W missense mutations are in the microtubule-binding region (red arrows) and are equivalent to the E237K and R286W mutations in human KIF5C (also see Fig. 2A,B). The *unc-116* loss-of-function allele, *e2310*, contains a *Tc5* transposon insertion after residue 692 in the stalk domain. (B) *C. elegans* contains six mechanosensory neurons. Specifically, the PLM neurons [PLM (L/R)] project a singular axon anteriorly that terminates (red arrow) before each ALM cell body. Image created with BioRender.com. (C) Example of normal axon termination in a wild-type PLM, where the axon terminates (arrow) before the ALM cell body (asterisk). (D) Example of PLM axon overextension phenotype in an *unc-116* loss-of-function mutant, where the axon terminates (arrow) past the ALM cell body (asterisk). (E) Loss-of-function mutations in *unc-116* cause axon termination defects. (F) Example of a wild-type PLM axon, where the axon tip (arrow) crosses the center of the vulva (asterisk) and terminates before the ALM cell body. (G) Example of an *unc-116* gain-of-function mutant PLM, where the axon tip (arrow) passes the PVM but fails to pass the center of the vulva (asterisk) before termination. (H) Early termination defects are caused by the *unc-116(rh24)* gain-of-function mutation, but not by the *unc-116* loss-of-function mutations. *n*=200 axons per genotype. PLM axons were visualized in L4 hermaphrodites with the *jsIs973* transgene that encodes *Pmec-7::rfp*. **P<0.01, ***P<0.0001 (two-tailed ‘N−1’ Chi-squared test for proportions). Error bars represent the standard error of the proportion. Scale bars: 10 µm.

To investigate a potential role for UNC-116 in axon targeting, we studied a series of mutations in *unc-116* (Fig. 1A) in the PLM, a mechanosensory neuron with its cell body in the animal's tail and an axon extending anteriorly along the lateral body wall. In nearly all wild-type animals, the PLM axon terminates before reaching the ALM cell body (Fig. 1B,C). Because of the reproducibility of PLM axon termination near this landmark, it is possible to sensitively assay for perturbations that cause either premature or late termination (Buddell et al., 2019). We first tested the effects of *unc-116* loss of function on PLM axon termination (Fig. 1C-E). We analyzed the hypomorphic *unc-116(e2310)* mutation, a Tc5 transposon insertion that disrupts the C-terminal tail of UNC-116 (Patel et al., 1993) (Fig. 1A). We found that the *unc-116(e2310)* mutation causes PLM axon overextension with a penetrance of 30% (Fig. 1E). We also tested the *unc-116(rh24sb79)* mutation, which is a hypomorph and revertant of the *unc-116(rh24)* gain-of-function mutation (Yang et al., 2005) (Fig. 1A). We found that the *unc-116(rh24sb79)* also causes PLM axon overextension with a penetrance of 28.5% (Fig. 1E). These observations suggest that UNC-116 can promote axon termination.

**Fig. 2.**
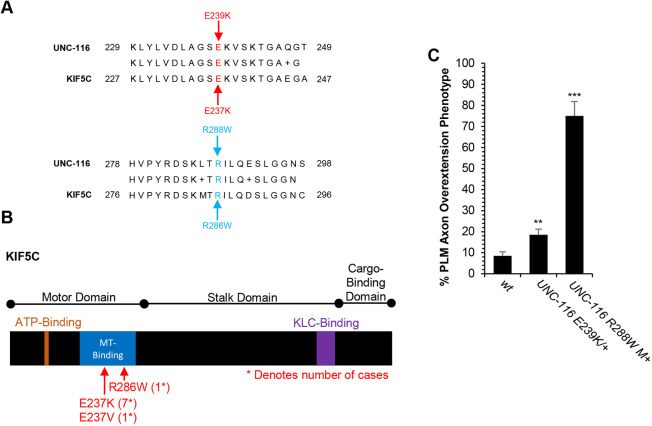
**Mutations equivalent to neurodevelopmental disorder-associated variants in the KIF5C kinesin-1 heavy chain cause PLM axon termination defects.** (A) The E239K mutation in UNC-116 is equivalent to the E237K mutation in KIF5C. The R288W mutation in UNC-116 is equivalent to the R286W mutation in KIF5C. (B) The KIF5C kinesin-1 heavy chain contains a motor domain, a stalk domain and a cargo-binding domain. Within the motor domain, there is an ATP-binding region and a microtubule-binding region. The stalk region contains a binding site for the kinesin-1 light chains (KLC). Three distinct *de novo* KIF5C variants are thought to be causative for a neurodevelopmental syndrome in humans: E237K (seven cases), E237V (one case) and R286W (one case). (C) UNC-116 E239K and R288W mutations cause PLM axon termination defects. UNC-116 E239K heterozygotes are *unc-116(syb4566)/hT2*. UNC-116 R288W homozygotes are the maternally rescued *unc-116(syb4669)* homozygous progeny of *unc-116(syb4669)/hT2* heterozygotes. *n*=200 for *unc-116(syb4566)* mutants. *n*=40 for *unc-116(syb4669)* mutants. PLM axons were visualized in L4 hermaphrodites with the *muIs32* transgene that encodes *Pmec-7::gfp*. **P<0.01, ***P<0.0001 (two-tailed ‘N−1’ Chi-squared test for proportions). Error bars represent the standard error of the proportion.

To further test the idea that UNC-116 can promote axon termination, we used the *unc-116(rh24)* gain-of-function mutation (Fig. 1A). If UNC-116 promotes axon termination, we would expect that the *unc-116* gain-of-function mutation would cause premature axon termination. In wild type, we found that PLM axons almost always terminate past the center of the vulva (Fig. 1A,F). However, in *unc-116(rh24)* gain-of-function mutants, we observed an early termination phenotype with a penetrance of 44.5%, where the PLM axon terminates before reaching the center of the vulva (Fig. 1G,H). By contrast, this early termination phenotype was rarely seen in the *unc-116(e2310)* or *unc-116(rh24sb79)* loss-of-function mutants (Fig. 1H). Although the *unc-116(rh24)* mutation mostly caused early axon termination, we note that in ∼16% of cases it caused PLM axon overextension (Fig. 1E). These observations are consistent with the idea that UNC-116 can promote axon termination.

### UNC-116 mutations equivalent to NDD-associated variants in the KIF5C KHC cause defects in PLM axon termination

The human ortholog of UNC-116 is known as KIF5C and *de novo* missense variants in this gene have been identified as candidate risk factors for NDDs (Wang et al., 2020). The strongest evidence comes from recurrent *de novo* variants that alter E237 of KIF5C and are associated with a neurodevelopmental syndrome that includes epilepsy, intellectual disability, autistic features and absent language (Cavallin et al., 2016; Michels et al., 2017; Poirier et al., 2013; Willemsen et al., 2014). The KIF5C E237K variant has been identified in seven individuals with this syndrome (Fig. 2A,B). In addition, the E237V variant has been identified in a single individual with this disorder. Moreover, other KIF5C *de novo* variants have been found in children with NDDs (Wang et al., 2020). For example, an R286W variant has been reported in a child with autism (Fig. 2A,B). Despite these findings, the effect of these KIF5C variants on neuronal development has yet to be tested. Moreover, as these are missense variants, their effect on neuronal development is difficult to accurately predict.

To determine how the KIF5C E237K and R286W variants might alter axon development, we used CRISPR/Cas9 to edit the *unc-116* gene and introduce the equivalent E239K and R288W mutations into the UNC-116 protein (Figs 1A, 2A). The *unc-116(E239K)* mutation was lethal without maternal rescue, precluding analysis of homozygotes. However, we found that in heterozygous *unc-116(E239K)/+* mutants, the PLM axon often failed to terminate normally and overextended past the ALM cell body (Fig. 2C). The *unc-16(R288W)* mutation was also lethal, but showed maternal rescue, allowing us to analyze maternally rescued homozygotes. We found that maternally rescued *unc-16(R288W)* mutants had PLM axon overextension with a penetrance of 75% (Fig. 2C). Together, these observations indicate that the E239K and R288W mutations in UNC-116 can disrupt axon termination. Moreover, they support the idea that the corresponding E237K and R286W variants in human KIF5C are likely to be causative for NDDs.

### Loss of the kinesin-1 cargo adaptor UNC-16 but not the KLCs causes axon termination defects

**Fig. 3.**
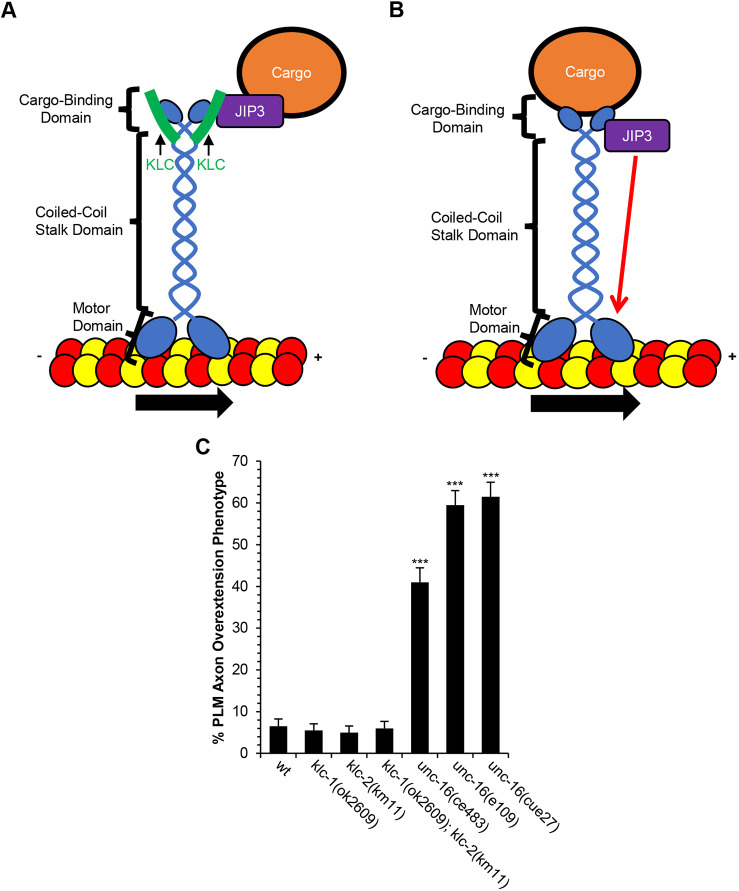
**Loss of UNC-16 (JIP3) function causes PLM axon termination defects.** (A) Illustration of cargo binding to kinesin. Kinesin-1 uses its light chains (KLC) to bind to kinesin adaptor JIP3 (UNC-16) to carry cargo. (B) Illustration of kinesin binding to cargo independent of its light chains. JIP3 binds to the C-terminus of kinesin and activates the motor activity by increasing motor velocity and run length (indicated by the red arrow). (C) Loss of UNC-16 function causes PLM axon termination defects. However, loss of KLC function does not affect axon termination. *n*=200 axons per allele. PLM axons were analyzed in L4 hermaphrodites using the *jsIs973* transgene that encodes *Pmec-7::rfp*. ***P<0.0001 (two-tailed ‘N−1’ Chi-squared test for proportions). Error bars represent the standard error of the proportion.

The most prevalent and best understood form of kinesin-1 is a tetramer that includes two KHCs and two KLCs. In this canonical form of kinesin-1, the KLCs bind to JNK-interacting proteins (JIPs), which in turn bind other cargo, such as vesicles and organelles (Fig. 3A) (Deng et al., 2014; Inagaki et al., 2001; Saez et al., 2020; Wang et al., 2011). Kinesin-1 can also function non-canonically, through a form that involves a dimer of KHC and the JIPs but omits the KLCs (Fig. 3B) (Cross et al., 2021; Lu et al., 2013; Palacios and St Johnston, 2002; Winding et al., 2016). However, relatively little is known about the functions of dimeric kinesin-1.

To investigate the contributions of these various kinesin-1 components to axon termination, we used mutations that disrupt the *C. elegans* UNC-16 JIP protein and both of the *C. elegans* KLCs. For these experiments, we used three different *unc-16* alleles to disrupt the function of the UNC-16 JIP protein and found that all caused substantial axon termination defects (Fig. 3C). To disrupt the KLCs we used the *klc-1(ok2609)* null allele and the *klc-2(km11)* hypomorphic allele. We found that axon termination defects were not caused by a *klc-1(ok2609)* mutation, a *klc-2(km11)* mutation, or by a *klc-1(ok2609); klc-2(km11)* double mutation (Fig. 3C). Although the *klc-2(km11)* allele is hypomorphic, we note that it causes defects in synaptic vesicle localization as well as defects in transport and localization of mitochondria in axons that are comparable in penetrance with that caused by the *unc-116(e2310)* allele (Sakamoto et al., 2005; Sure et al., 2018). Therefore, these observations suggest that the role of kinesin-1 in axon termination may not require the KLCs.

### UNC-16 and UNC-116 function with NEKL-3 to promote axon termination

To investigate the mechanism of UNC-16 in axon termination, we sought to identify proteins that function with UNC-16 to promote axon termination. We considered the NEKL-3 kinase as a candidate because ongoing work in our laboratory had suggested that NEKL-3 is required for PLM axon termination. Moreover, the NEK6 and NEK7 orthologs of NEKL-3 have been implicated in the regulation of microtubule stability during mitosis in the HeLa cell line (Cohen et al., 2013; Fry et al., 2012; Kim et al., 2007; Yissachar et al., 2006). As microtubule stability is also important for PLM axon termination (Borgen et al., 2017), we reasoned that NEKL-3 might also be involved in this process. Further supporting this hypothesis, NEK7 functions in cultured mouse neurons to regulate microtubule dynamics and axonal arborization (Hinojosa et al., 2018).

**Fig. 4.**
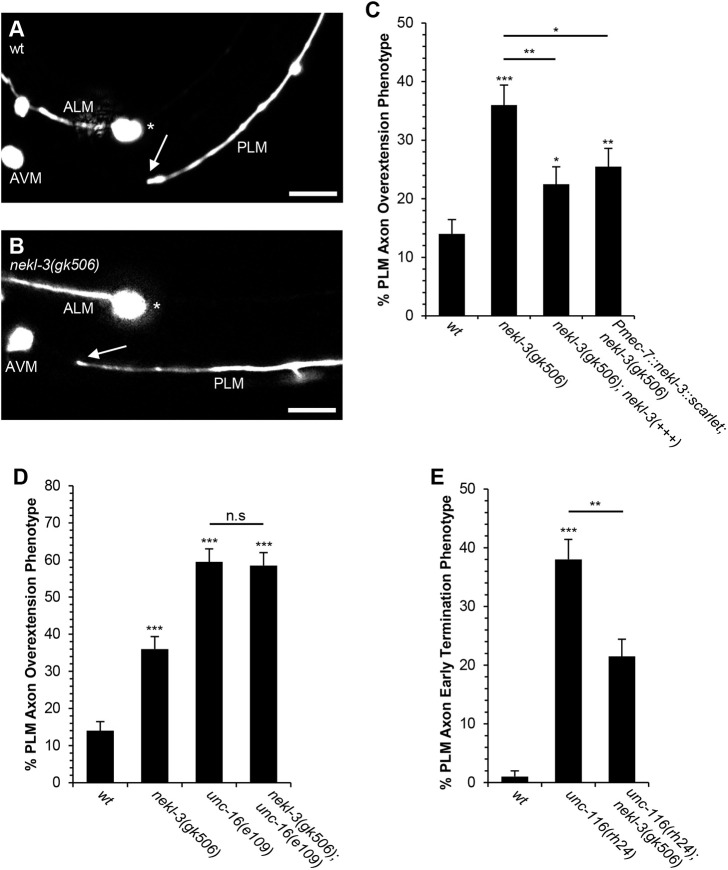
**NEKL-3 functions with UNC-16 and UNC-116 to promote axon termination.** (A) Example of wild-type PLM axon, where the axon terminates before reaching the ALM cell body. (B) Example of a *nekl-3(gk506)* mutant PLM axon, where the PLM axon terminates past the ALM cell body (arrow). The asterisk indicates the ALM cell body. Scale bars: 10 µm. (C) The *nekl-3(gk506)* null mutation causes PLM axon termination defects. These defects are partially rescued by the *Pmec-7::nekl-3::scarlet* transgene, which is specifically expressed in PLM and other touch neurons. These defects were also partially rescued by the *mnEx174* transgene, which includes a cosmid that contains *nekl-3* and its upstream regulatory region. (D) The *nekl-3* gene functions in a genetic pathway with *unc-16* to promote PLM axon termination. The penetrance of axon termination defects was not enhanced in *nekl-3; unc-16* double null mutants relative to *unc-16* single null mutants. (E) The PLM axon early termination phenotype caused by the *unc-116(rh24)* mutation is partially suppressed in the *nekl-3(gk506); unc-116(rh24)* double mutant. *n*=100 axons for wild-type early termination; *n*=200 axons for all other genotypes. PLM axons were analyzed in the late L1 stage using the *jsIs973* transgene that encodes *Pmec-7::rfp.* The *nekl-3(gk506)* mutation is lethal and was maintained with the *mnEx174* rescuing cosmid. Axons were scored in *nekl-3(gk506)* homozygotes that had lost the *mnEx174* rescuing cosmid. The *mnEx174* transgene encodes the F19H6 cosmid, which includes the *nekl-3* gene, and the pTG96 *Psur-5::gfp* co-injection marker. The *Pmec-7::nekl-3::scarlet* transgene is *CueSi33*, which is integrated into the *jsTi1493* landing pad on chromosome IV. *P<0.05, **P<0.01,***P<0.0001 (two-tailed ‘N−1’ Chi-squared test for proportions). Asterisks above bars indicate statistically significant differences relative to wild type and asterisks above brackets indicate statistically significant differences between two groups. n.s, no significant difference between two groups. Error bars represent the standard error of the proportion.

To investigate a potential role for NEKL-3 in axon termination, we examined PLM axon termination in *nekl-3(gk506)* null mutants at the late L1 stage. The *nekl-3(gk506)* allele is lethal and is maintained with an extrachromosomal array that contains copies of the *nekl-3* gene. We analyzed *nekl-3(gk506)* homozygotes that had lost the extrachromosomal copies of the *nekl-3* gene and found PLM axon termination defects with a penetrance of 36% (Fig. 4A-C). We also found that these PLM axon termination defects could be partially rescued by the extrachromosomal copies of the *nekl-3* gene (Fig. 4C). To determine whether NEKL-3 functions cell-autonomously to regulate PLM axon termination, we used the *mec-7* promoter to transgenically express NEKL-3::SCARLET in the touch receptor neurons, including the PLM. We found that the axon termination defects caused by the *nekl-3(gk506)* null allele were partially rescued by the *Pmec-7::nekl-3::scarlet* transgene (Fig. 4C). The partial nature of this rescue could be the result of suboptimal levels or timing of NEKL-3::SCARLET expression. Together, these observations suggest that NEKL-3 functions cell autonomously to promote PLM axon termination. Moreover, these observations also indicate that the NEKL-3::SCARLET fusion protein is functional in axon termination.

To test the idea that UNC-16 functions with NEKL-3 to promote axon termination, we conducted genetic analysis to determine whether the *unc-16* and *nekl-3* genes function in a genetic pathway. If two genes function in a genetic pathway, it is expected that the penetrance of defects in the double mutant will not be enhanced relative to the single mutant with the greatest penetrance. For this experiment, we analyzed PLM axon termination defects in *unc-16(e109)* mutants and *unc-16(e109); nekl-3(gk506)* double mutants at the late L1 stage. We found that *unc-16(e109); nekl-3(gk506)* double null mutants had a penetrance that was not enhanced relative to *unc-16(e109)* single null mutants (Fig. 4D), suggesting that *unc-16* functions in a genetic pathway with *nekl-3* to promote PLM axon termination.

To test the idea that NEKL-3 functions with UNC-116, we attempted, unsuccessfully, to construct an *unc-116(e2310); nekl-3(gk506)* double mutant strain. As an alternative, we used the *unc-116(rh24)* gain-of-function allele to ask whether the early axon termination phenotype caused by this mutation can be suppressed by the loss of *nekl-3* function. Consistent with this prediction, we found that the penetrance of early axon termination in *unc-116(rh24); nekl-3(gk506)* double mutants was significantly reduced relative to *unc-116(rh24)* single mutants (Fig. 4E)*.* This observation indicates that the early termination defects caused by the *unc-116(rh24)* allele are dependent on NEKL-3 function. Moreover, this observation supports a model whereby UNC-116 functions upstream of NEKL-3 to promote axon termination.

### UNC-16 and UNC-116 promote axonal localization and motility of NEKL-3

**Fig. 5.**
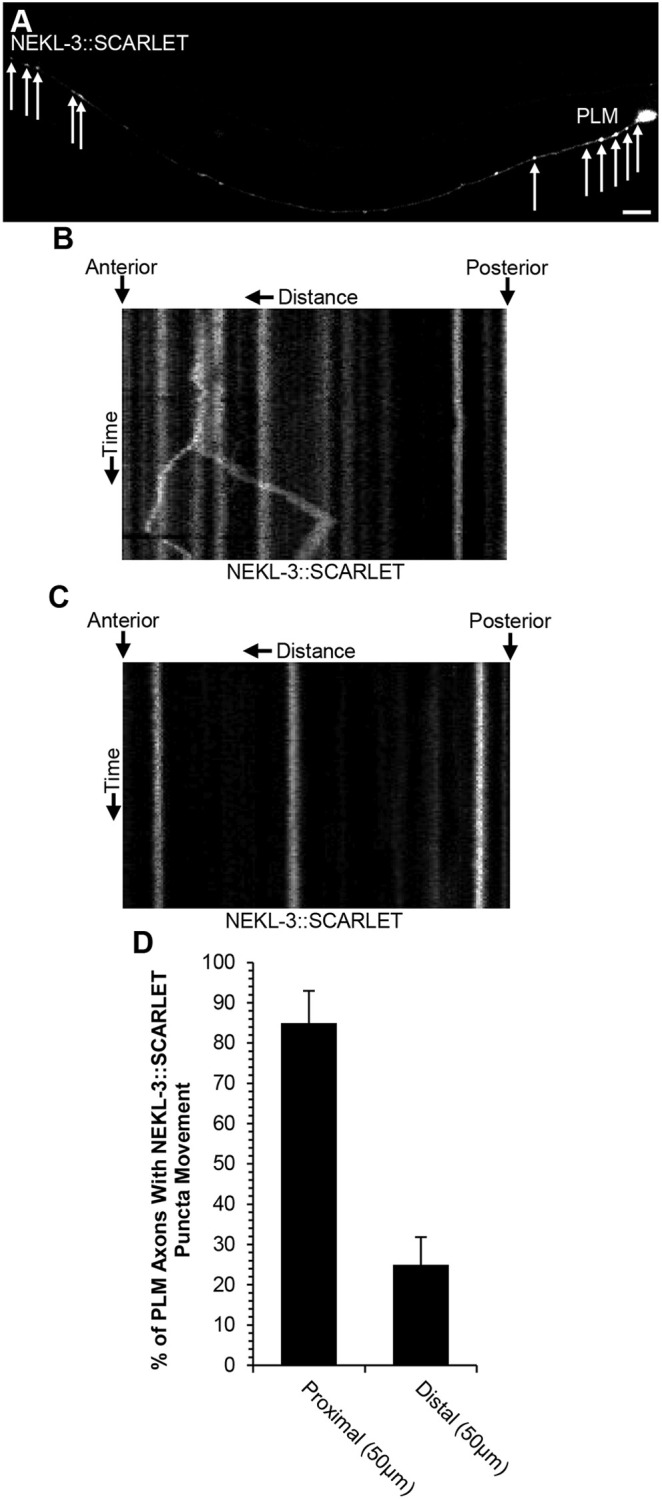
**NEKL-3 moves bidirectionally along the PLM axon.** (A) Example of NEKL-3::SCARLET puncta in a wild-type PLM axon. Arrows indicate NEKL-3::SCARLET puncta in the proximal and distal 50 µm regions. (B) Example kymograph illustrating puncta movement in a wild-type PLM proximal axon (50 µm closest to the cell body). Arrows denote movement of NEKL-3::SCARLET puncta along the PLM axon. (C) Example kymograph illustrating puncta movement in a wild-type PLM distal axon (50 µm closest to the axon tip). Distance is from 0 to 50 µm along the *x*-axis. Time is from 0 min to 2 min along the *y*-axis. (D) Percent of NEKL-3::SCARLET puncta with movement in the distal and proximal PLM axon. *n*=20 axons for the proximal axon; 40 axons for the distal axon. NEKL-3::SCARLET was expressed by the *CueEx31* transgene that encodes *Pmec-7::nekl-3::scarlet*. PLM axons were visualized with the *muIs32* transgene that encodes *Pmec-7::*gfp.

To further investigate the role of NEKL-3 in axon termination, we conducted imaging studies of NEKL-3::SCARLET in the PLM axon. We found that NEKL-3::SCARLET localizes to puncta throughout the PLM axon (Fig. 5A). To further explore the function of NEKL-3 we also conducted time-lapse imaging on PLM axons expressing NEKL-3::Scarlet (Fig. 5B-D; Movies 1-3). We observed bidirectional transport of NEKL-3::SCARLET puncta (Fig. 5B; Movies 1-3). We also noticed a marked difference in NEKL-3 motility between the proximal axon (50 µm closest to cell body) and distal axon (50 µm closest to the axon tip) segments (Fig. 5B-D). We found that 85% of proximal axon segments exhibited motile SCARLET puncta. By contrast, only 25% of distal axon segments exhibited motile NEKL-3::Scarlet puncta. These observations suggest that NEKL-3 undergoes fast bidirectional transport within the proximal axon but is more stable within the distal axon.

**Fig. 6.**
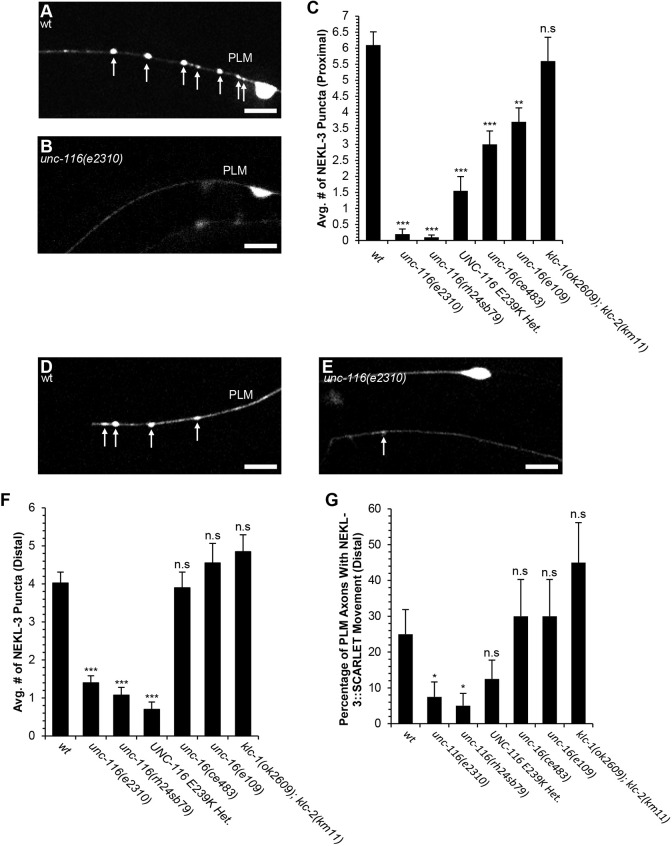
**Loss of UNC-116 or UNC-16 function decreases NEKL-3 puncta in the PLM axon.** (A) Example of NEKL-3::SCARLET puncta in the proximal axon of a wild-type (wt) PLM. (B) Example of NEKL-3::SCARLET puncta in the proximal axon of an *unc-116(e2310)* mutant PLM. (C) Loss-of-function mutations in *unc-16* or *unc-116* cause a significant decrease in NEKL-3::SCARLET puncta in the PLM proximal axon. (D) Example of NEKL-3::SCARLET puncta in the distal axon of a wild-type PLM. (E) Example of NEKL-3::SCARLET puncta in the distal axon of an *unc-116(e2310)* mutant. (F) Loss of UNC-116 function causes a significant decrease in NEKL-3::SCARLET puncta in the PLM distal axon. However, loss of UNC-16 function does not affect NEKL-3::SCARLET puncta in the PLM distal axon. (G) Loss of UNC-116 function causes a significant decrease in the motility of NEKL-3::SCARLET puncta in the PLM distal axon. However, loss of UNC-16 function does not affect the motility of NEKL-3::SCARLET puncta in the PLM distal axon. For analysis of proximal punta, *n*=20 for all genotypes. For analysis of distal puncta, *n*=40 for all genotypes except the *unc-16* alleles, where *n*=20. NEKL-3::SCARLET puncta were expressed by the *CueEx31* transgene that encodes *Pmec-7::nekl-3::scarlet*. Arrows indicate NEKL-3::SCARLET puncta. In C and F, **P<0.01, ***P<0.0001 (unpaired two-tailed *t*-test with unequal variances) and error bars represent the standard error of the mean. In G, *P<0.05 (two-tailed ‘N−1’ Chi-squared test for proportions) and error bars represent the standard error of the proportion. n.s, no significant difference. Scale bars: 10 µm.

Considering the fast transport of NEKL-3 in the PLM axon, we asked whether UNC-116 is required for the normal motility or localization of NEKL-3 in the axon. In wild-type PLM axons we found that the proximal axon (50 µm closest to cell body) contained an average of 6.1±0.4 NEKL-3::SCARLET punta (mean±s.e.m.; Fig. 6A,C). By contrast, NEKL-3::SCARLET puncta were almost completely absent from the proximal axon in *unc-116(e2310)* mutants (Fig. 6B,C). In the distal axon (50 µm closest to the axon tip) of *unc-116(e2310)* mutants, we found a 65% reduction in puncta number relative to wild type (Fig. 6D-F). Moreover, we found that the *unc-116(e2310)* mutation also caused a decrease in the proportion of distal axon segments that display motile NEKL-3::SCARLET puncta (Fig. 6G). We repeated these experiments with the *unc-116(rh24sb79)* loss-of-function mutation and found similar results (Fig. 6C,F,G). Moreover, we found that the heterozygous UNC-116(E239K) mutation also reduced the number of NEKL-3::SCARLET punta in the proximal and distal axons (Fig. 6C,F). The UNC-116(E239K) mutation also reduced NEKL-3 motility in the distal axon, however this did not reach statistical significance (Fig. 6G). By contrast, we found no significant change in the number of NEKL-3::SCARLET puncta in *klc-1(ok2609); klc-2(km11)* double mutants, consistent with our finding that this double mutant does not affect PLM axon termination (Fig. 6C,F,G). These observations suggest that UNC-116 is required for the normal distribution and motility of NEKL-3 in the PLM axon.

To further investigate the role of UNC-116 and NEKL-3 in axon termination, we also observed the localization of NEKL-3::SCARLET in *unc-116(rh24)* gain-of-function mutants. As described above, this allele mostly produces early PLM axon termination defects but can sometimes produce PLM axon overextension defects. Therefore, we separately analyzed NEKL-3:SCARLET puncta in *unc-116(rh24)* mutants with early PLM termination and in *unc-116(rh24)* mutants with overextended PLM axons (Table 1). In the distal axon of mutants with early termination, we found that the number of NEKL-3::SCARLET puncta was unchanged relative to wild type. On the other hand, the number of NEKL::SCARLET puncta in the proximal axon of these mutants was significantly decreased relative to wild type. One interpretation of these results is that UNC-116 gain-of-function moves NEKL-3 puncta out of the proximal axon and that the function of NEKL-3 in the distal axon could be increased independently of puncta number. We also analyzed NEKL-3::SCARLET puncta in *unc-116(rh24)* mutants that had PLM overextension defects. In these mutants, we found that the number of NEKL-3::SCARLET puncta was reduced relative to wild type in both the distal and proximal axon segments (Table 1). This observation is consistent with our findings that a reduction in NEKL-3 function is associated with axon overextension.

**Table 1.**
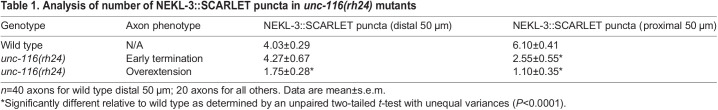
Analysis of number of NEKL-3::SCARLET puncta in *unc-116(rh24)* mutants

As our genetic data suggest that UNC-16 functions with NEKL-3 to promote axon termination, we also asked whether UNC-16 is required for localization of NEKL-3 in the PLM axon. We found that both the *unc-16(ce483)* and *unc-16(e109*) loss-of-function mutations caused a reduction of the number of NEKL-3::SCARLET puncta in the PLM proximal axon (Fig. 6C). By contrast, these *unc-16* loss-of-function mutations did not affect the number of NEKL-3::SCARLET puncta in the PLM distal axon (Fig. 6F). Likewise, these *unc-16* loss-of-function mutations did not alter the proportion of distal axon segments that display motile NEKL-3::SCARLET puncta (Fig. 6G). These observations suggest that UNC-16 promotes localization of NEKL-3::SCARLET in the proximal axon but does not affect NEKL-3::SCARLET in the distal axon.

### NEKL-3 functions with the RPM-1 pathway to promote axon termination

**Fig. 7.**
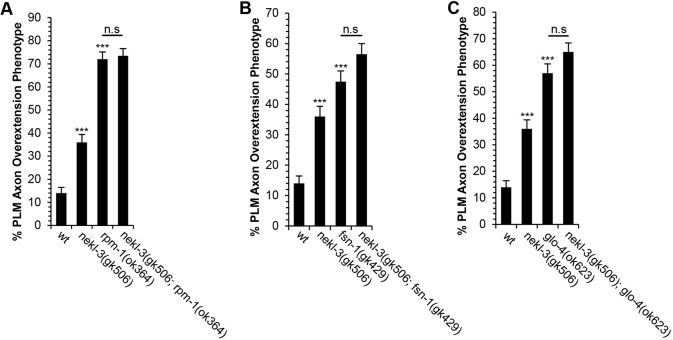
**NEKL-3 functions with the RPM-1 pathway to promote axon termination.** (A) Null mutations in *nekl-3* and *rpm-1* (*gk506* and *ok364*, respectively) cause PLM axon termination defects in the late L1 stage. However, PLM axon termination defects are not enhanced in *nekl-3(gk506); rpm-1(ok364)* double mutants. (B) Null mutations in *nekl-3* and *fsn-1* (*gk506* and *gk429*, respectively) cause PLM axon termination defects in the late L1 stage. However, PLM axon termination defects are not enhanced in *nekl-3(gk506); fsn-1(gk429)* double mutants. (C) Null mutations in *nekl-3* and *glo-4* (*gk506* and *ok623*, respectively) cause PLM axon termination defects in the late L1 stage. However, PLM axon termination defects are not enhanced in *nekl-3(gk506); glo-4(ok623)* double mutants. *n*=200 axons per genotype. PLM axons were analyzed using the *jsIs973* transgene that encodes *Pmec-7::rfp*. ***P<0.0001 (two-tailed ‘N−1’ Chi-squared test for proportions). n.s, no significant difference relative to control. Error bars represent the standard error of the proportion.

RPM-1 and its ortholog Phr1 (Mycbp2) are ubiquitin ligases that can regulate axon termination (Schaefer et al., 2000; Zhen et al., 2000). The role of these proteins in axon termination are due, in part, to their ability to regulate microtubule stability (Borgen et al., 2017; Hendricks and Jesuthasan, 2009; Lewcock et al., 2007). Considering that the NEK6 and NEK7 orthologs of NEKL-3 are also regulators of microtubule stability (Adib et al., 2019; Cohen et al., 2013; Freixo et al., 2018), we wanted to determine the relationship between NEKL-3 and RPM-1 in the regulation of axon termination. For these experiments, we used the *rpm-1(ok364)* allele and the *nekl-3(gk506)* allele, both of which are thought to be null alleles (Park and Rongo, 2018; Yochem et al., 2015). We found that the penetrance of axon termination defects in *nekl-3(gk506); rpm-1(ok364)* double mutants was not significantly enhanced relative to *rpm-1(ok364)* single mutants. (Fig. 7A). These data suggest that *rpm-1* functions in the same pathway as *nekl-3* to promote axon termination.

As we found that NEKL-3 functions with RPM-1, we next wanted to determine whether it also functions with FSN-1 and GLO-4, two key interaction partners for RPM-1 (Grill et al., 2007). To determine how *nekl-3* functions with the genes that encode the FSN-1 and GLO-4 proteins, we constructed double mutants between *nekl-3(gk506)* and null alleles for *fsn-1* and *glo-4*. We found that the penetrance of axon termination defects in *nekl-3(gk506); fsn-1(gk429)* was no greater than the penetrance of axon termination defects in *fsn-1(gk429)* single mutants (Fig. 7B). Likewise, we also found that the penetrance of axon termination defects in *nekl-3(gk506); glo-4(ok623)* was no greater than the penetrance of axon termination defects in *glo-4(ok623)* single mutants (Fig. 7C). Together, these observations suggest that *nekl-3* functions in a genetic pathway that includes *rpm-1*, *fsn-1* and *glo-4*.

## DISCUSSION

Previous work has identified a variety of roles for UNC-116 and UNC-16 in mediating the retrograde and anterograde transport of various cargo within the axon. However, the mechanisms through which axonal transport of specific cargo can control axon development remains poorly understood. Here, we describe a novel mechanism for the control of axon termination that involves UNC-116, UNC-16 and the NEKL-3 kinase. We propose a model where UNC-116 and UNC-16 promote localization of the NEKL-3 in the axon. Our genetic data also suggest that the NEKL-3 kinase functions with the RPM-1 ubiquitin ligase to control axon termination. An understanding of this mechanism may promote our understanding of how genetic disruptions in the *KIF5C* and *JIP3* genes can lead to NDDs.

### Roles of kinesin-1 in neuronal development

The best-known functions of kinesin-1 in neuronal development involve its tetrameric form that includes two heavy chains and two light chains. For example, in axon development, the KHCs and KLCs function together to transport CRMP2 into the nascent axon, where it functions with WAVE1 (WASF1) to promote polarization (Inagaki et al., 2001; Kawano et al., 2005; Kimura et al., 2005). Likewise, the KHCs and KLCs also function together to transport Rab-10-containing vesicles into the axon (Deng et al., 2014; Wang et al., 2011). Later in axon development, the KHCs and KLCs can function together to transport CB1 receptors in the axon, thereby promoting axon growth in response to cannabinoids (Saez et al., 2020). Kinesin-1 also functions with its light chains to regulate the development and function of synapses. (Hoerndli et al., 2013; Sakamoto et al., 2005).

A less understood aspect of kinesin-1 function involves the KHCs functioning independently of the KLCs. For example, the KHC can function with the aTm1 cargo adaptor to mediate mRNA localization independently of the KLCs (Cross et al., 2021; Palacios and St Johnston, 2002). Likewise, the KHCs can bind directly to microtubules and transport them within axons and dendrites independently of the KLCs (Lu et al., 2013; Winding et al., 2016). This process is known as microtubule sliding and is thought to promote the initial protrusion of the nascent axon as well as axodendritic polarity. Moreover, in *Drosophila* neurons the KHC can transport mitochondria independently of the KLCs (Glater et al., 2006). Taken together, these observations suggest that the KHC can form a homodimer that binds directly to certain cargo molecules.

Our results reveal a novel role for the KHCs in axon termination and suggest that this process might occur independently of the KLCs. We report that a loss-of-function mutation in the *unc-116* KHC gene causes axon termination defects. However, single, or double loss-of-function mutations in the *klc-1* and *klc-2* KLC genes do not cause axon termination defects. One caveat of this analysis is that the *klc-2* allele is hypomorphic. However, this *klc-2(km11)* hypomorphic allele causes defects in the localization of synaptic vesicles and mitochondria that are comparable with those caused by the *unc-116(e2310)* allele (Sakamoto et al., 2005; Sure et al., 2018). Despite its effect on synaptic vesicles and mitochondria, the *klc-2(km11)* allele does not cause any defects in axon termination. Together, these observations suggest that defects in the localization of synaptic vesicles and mitochondria do not underlie the axon termination defects that are caused by loss of *unc-116* function. Moreover, they also suggest that the role of UNC-116 in axon termination might not involve the KLCs.

### Role of UNC-16 in axon transport and termination of axon growth

The best-known function of UNC-16 involves retrograde transport of vesicles and organelles. For example, UNC-16 binds directly to the retrograde motor dynein and can mediate the retrograde transport of APL-1 (Arimoto et al., 2011). Moreover, loss of UNC-16 function causes a dramatic increase in the number of endosomes, lysosomes, and mitochondria within axons, suggesting that UNC-16 promotes retrograde clearance of vesicles and organelles from axons (Celestino et al., 2022; Sure et al., 2018). Likewise, UNC-16 can promote retrograde transport and clearance of autophagosomes from synapses (Hill et al., 2019).

A less studied aspect of UNC-16 function involves anterograde transport. The role of UNC-16 in anterograde transport occurs as a result of its binding to kinesin-1. For example, dynein can be transported anteriorly by interaction with kinesin-1 and UNC-16 (Arimoto et al., 2011). Moreover, in rat neurons TrkB can be transported anteriorly into distal axons through a direct interaction with JIP3 (Huang et al., 2011; Sun et al., 2017). Consistent with the ability of UNC-16 to promote anterograde and retrograde transport, sciatic nerve ligation experiments have indicated that JIP3 is associated with vesicles moving in both the anterograde and retrograde directions (Abe et al., 2009).

We report a novel role for UNC-16 in axon development that is consistent with anterograde transport of the NEKL-3 kinase. We find that UNC-16 is required for axon termination and that loss of UNC-16 decreases the number of NEKL-3 puncta in the proximal axon. Together, these observations suggest that UNC-16 can promote transport of NEKL-3 into the proximal axon. However, it is surprising that loss of UNC-16 function does not alter the number of NEKL-3 puncta in the distal axon. One possible explanation for this observation is that *unc-16* mutants decrease the activity of NEKL-3 puncta in the distal axon without altering the number of puncta. In this regard, we note that in the proximal axon NEKL-3 is motile, whereas in the distal axon it is stable. We speculate that motile NEKL-3 in the proximal axon could be important to deliver new NEKL-3 molecules to the distal axon. If this proximal motility is compromised, the stable NEKL-3 in the distal axon could become older and less active.

### Role of NEKL-3 in axon termination

A key question for future investigations will be to determine how NEKL-3 promotes axon termination. As a starting point, our genetic data suggest that NEKL-3 can function with RPM-1 to promote axon termination. Several studies in multiple systems have indicated that RPM-1 and its orthologs can promote axon termination by destabilizing microtubules. For example, genetic studies have indicated that RPM-1 signaling destabilizes microtubules to cause PLM growth cone collapse and axon termination in *C. elegans* (Borgen et al., 2017). Consistent with these findings, the PHR1 ortholog of RPM-1 (Mycbp2) also destabilizes microtubules to promote axon targeting in zebrafish (Hendricks and Jesuthasan, 2009). Moreover, loss of PHR1 function causes an excess of disorganized and misoriented microtubules within the growth cones of mice and zebrafish (Hendricks and Jesuthasan, 2009; Lewcock et al., 2007). Furthermore, biochemical fractionation studies have indicated that PHR1 is associated with microtubules (Lewcock et al., 2007). Thus, we hypothesize that NEKL-3 promotes termination of axon growth by functioning with RPM-1 to destabilize microtubules in the growth cone.

Consistent with the idea that NEKL-3 can function with RPM-1 to destabilize microtubules in the growth cone, studies of the NEK7 ortholog of NEKL-3 in HeLa cells and cultured neurons have suggested that NEK7 can destabilize microtubules. For example, in HeLa cells, knockdown of NEK7 reduces the rate of microtubule growth and time spent in catastrophe, suggesting that the microtubules are stabilized in the absence of NEK7 (Cohen et al., 2013). Moreover, in cultured mouse neurons, loss of NEK7 can reduce the rate of microtubule growth, also consistent with the stabilization of microtubules (Hinojosa et al., 2018). Together these observations suggest that NEKL-3 can destabilize microtubules and are consistent with the hypothesis that NEKL-3 and RPM-1 function together to promote axon termination by destabilizing microtubules in the growth cone.

### Support for a role of KIF5C variants in NDDs

Candidate risk genes for NDDs are typically identified when a higher-than-expected rate of *de novo* variants in a particular gene is detected within a population of affected children. A key challenge in interpreting these studies is to determine how likely a given *de novo* mutation is to affect neuronal development. This is particularly challenging with missense mutations, where an algorithm is used to predict the likelihood that a given *de novo* missense mutation will affect protein function. Thus, a key goal in validating candidate genes is to determine whether *de novo* variants from affected individuals can actually disrupt neuronal development. In the case of *KIF5C*, four missense *de novo* variants have been identified along with a single protein-truncating variant (Wang et al., 2020). Our genetic analysis indicates that at least two of these missense variants can disrupt neuronal development, thereby increasing confidence that *de novo* variants in *KIF5C* gene can be causative for NDDs.

Our study also provides insight into how *KIF5C* variants might cause NDDs. Kinesin-1 can regulate synaptic plasticity by mediating the trafficking of AMPA and GABA receptors (Brachet et al., 2021; Hoerndli et al., 2013; Nakajima et al., 2012). Thus, it is reasonable to hypothesize that KIF5C variants cause NDDs by disrupting synaptic plasticity. Although our results do not contradict this hypothesis, they do suggest that *KIF5C* variants can also cause NDDs by disrupting axon targeting. Moreover, this disruption in axon targeting may occur independently of any alteration in synaptic function that might also occur. Future research into how the *KIF5C* variants alter the development and function of mammalian neurons may help to clarify the mechanisms that give rise to NDDs.

## MATERIALS AND METHODS

### Mutant alleles and transgenes

*C. elegans* strains were maintained at 20°C on nematode growth medium (NGM)-agar plates using standard procedures. All experiments were performed on hermaphrodites. Alleles used in the study include: wild-type N2, *unc-116(syb4566))/hT2* [also known as *unc-116(E239K)*], *unc-116(syb4669)/hT2* [also known as *unc-116(R288W)*], *unc-116(e2310)*, *unc-116(rh24sb79)*, *klc-1(ok2609)*, *klc-2(km11)*, *unc-116(rh24)*, *nekl-3(gk506) mnEx174* [*Pnekl-3::nekl-3*], *rpm-1(ok364)*, *fsn-1(gk429)*, *glo-4(ok623)*, *unc-16(e109)* and *unc-16(ce483).* Double mutants were constructed using standard methods. Briefly, male worms were placed with hermaphrodites and allowed to mate. Mutations were selected in subsequent generations by phenotype or by PCR assay. The *unc-116(syb4566)* and *unc-116(syb4669)* mutant alleles were obtained from SunyBiotech. The *unc-116(rh24sb79)* mutant allele was obtained from Paul Mains (University of Calgary, Canada). The *nekl-3(gk506)* allele was obtained from David Fay (University of Wyoming, WY, USA). The *jsIs973* transgene was obtained from Michael Nonet (Washington University in St. Louis, MO, USA). The *cueEx31* transgene was created by injecting a mixture of DNA plasmids encoding *Pmec-7::nekl-3::scarlet* (2 ng/µl), Podr*-1::rfp* (50 ng/µl) along with an empty pBluescript vector (50 ng/µl). *Pmec-7::nekl-3::scarlet* plasmid was created by PCR and Gibson assembly. The *Podr-1::rfp* plasmid was obtained from Cori Bargmann (Rockefeller University, NY, USA). The *CueSi33* transgene is a single copy insertion of *Pmec-7::nekl-3::scarlet::unc-54* 3′ untranslated region that was created by recombination-mediated cassette exchange using the pLF3FShC integration vector (Addgene plasmid #153083) and the jsTi1493 landing pad on chromosome IV (Nonet, 2020). The *Pmec-7::nekl-3::scarlet::unc-54* plasmid was synthesized by Twist Bioscience. All other mutants and transgenic strains were obtained from the *Caenorhabditis* Genetics Center (CGC).

### Analysis of phenotypes

To determine the PLM axon termination phenotypes, *C. elegans* were randomly picked from plates and mounted on a 5% agarose pad, anesthetized with levamisole, and observed with a 40× objective on a Zeiss Axio Imager M2 microscope. PLM axons were observed using the *jsIs973* and *muIs32* transgenes, which encode RFP and GFP fluorescent proteins, respectively, and use an *mec-7* promoter to drive expression of these proteins in all mechanosensory neurons. A PLM axon was considered overextended if the axon tip terminated anteriorly to the ALM cell body. A PLM axon was considered to terminate early if the axon tip did not cross the center of the vulva. Data for each genotype was collected from two or more generations and all data collected were included in our analysis.

For analysis of axon phenotypes in lethal mutants, we used genetic balancers and rescuing transgenes to maintain the mutations. The *unc-116(R288W)* and the *unc-116(E239K)* mutations were maintained over the hT2 balancer. For the *unc-116(R288W)* mutation, we analyzed homozygous progeny that had been rescued by a maternal contribution from heterozygous parents. For the *unc-116(E239K)* mutation, we were unable to obtain maternally rescued homozygotes, and therefore we analyzed *unc-116(E239K)*/hT2 heterozygotes. The *nekl-3(gk506)* mutation is lethal and was maintained as a homozygous strain rescued by an extrachromosomal *Pnekl-3::nekl-3* transgene and progeny that had lost the transgene were analyzed.

### Imaging of NEKL-3::SCARLET

To observe localization of NEKL-3::SCARLET in the PLM axon we used the *cueEx31* transgene, which encodes *Pmec-7::nekl-3::scarlet.* The number and motility of NEKL-3 puncta were analyzed by mounting L4 stage *C. elegans*, anesthetized with levamisole, on a 5% agarose pad and observing with a 40× water objective on a Nikon ECLIPSE Ti microscope equipped with the X-Light V2 L-FOV spinning disk system within 30 min of levamisole application. To determine the percentage of PLM axons with movement of NEKL-3, videos were recorded using the Nikon Elements program at 40× magnification on the spinning disk confocal microscope. Videos were created by imaging a three-layered *z*-stack every 10 s for 2 min total. Puncta size and movement were analyzed using ImageJ software. Only puncta that were greater than 0.6 µm were analyzed. A punctum was considered motile when it moved anteriorly, posteriorly or in both directions at any time during the 2 min recording period. A PLM axon segment (50 µm segment closest to the cell body or 50 µm segment closest to the axon tip) was considered to have movement if at least one NEKL-3::SCARLET punctum within the segment moved within 2 min. To generate kymographs, videos were made by recording a single-layered image at 1 s intervals for 2 min total. Kymographs were generated using ImageJ software.

### Statistics

Significant PLM axon termination defects were analyzed using the ‘N−1’ Chi-squared test at a 95% confidence interval (CI) to compare with the control. We analyzed 200 axons for each allele unless otherwise stated. To determine significant differences among strains with *Pmec7::nekl-3::scarlet* expression, an unpaired two-tailed independent samples *t*-test was used at a 95% CI.

## Supplementary Material

10.1242/develop.201654_sup1 Supplementary information
