# Supplementary material for: UNC-116 and UNC-16 function with the NEKL-3 kinase to promote axon targeting
Source: Development. 2023 Sep 27;150(18):dev201654. doi: 10.1242/dev.201654 (PMC10561693; doi:10.1242/dev.201654)
Supplement: Supplementary information [file develop-150-201654-s1.pdf]

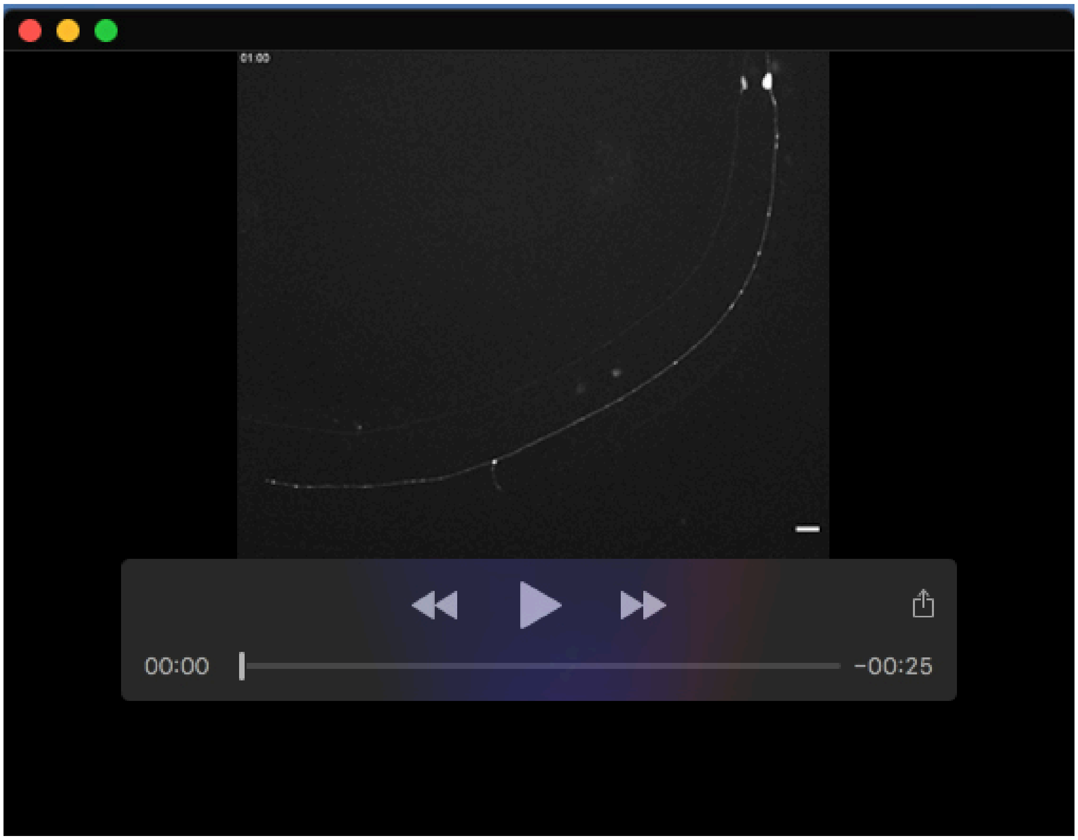

Movie 1. NEKL-3 movement across the PLM axon. NEKL-3 moves bidirectionally in the proximal end of the PLM axon and becomes more stable in the distal end. NEKL-3::SCARLET puncta were analyzed using the *Pmec-7::nekl-3::scarlet* transgene that expresses NEKL-3::SCARLET in touch receptor neurons. A three-layered Z-stack (0.5μm steps) was taken at ten second intervals for two minutes total with a 40x water objective on a spinning disk confocal microscope. Scale bar represents 10μm.

Development • Supplementary information

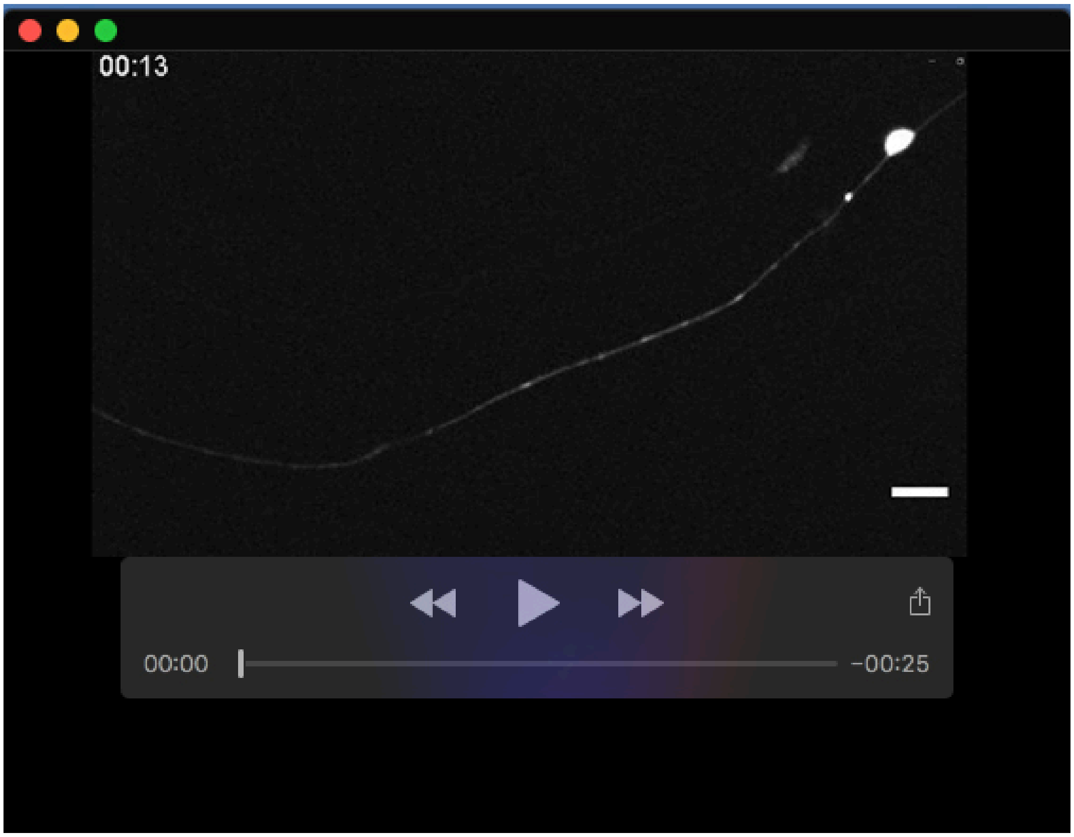

Movie 2. NEKL-3 movement in the proximal end of the PLM axon. NEKL-3 moves bidirectionally in the proximal region of the PLM axon. NEKL-3::SCARLET puncta were analyzed using the *Pmec-7::nekl-3::scarlet* transgene that expresses NEKL-3::SCARLET in touch receptor neurons. An image was taken at one second intervals for two minutes total with a 40x water objective on a spinning disk confocal microscope. Scale bar represents 10μm.

Development • Supplementary information

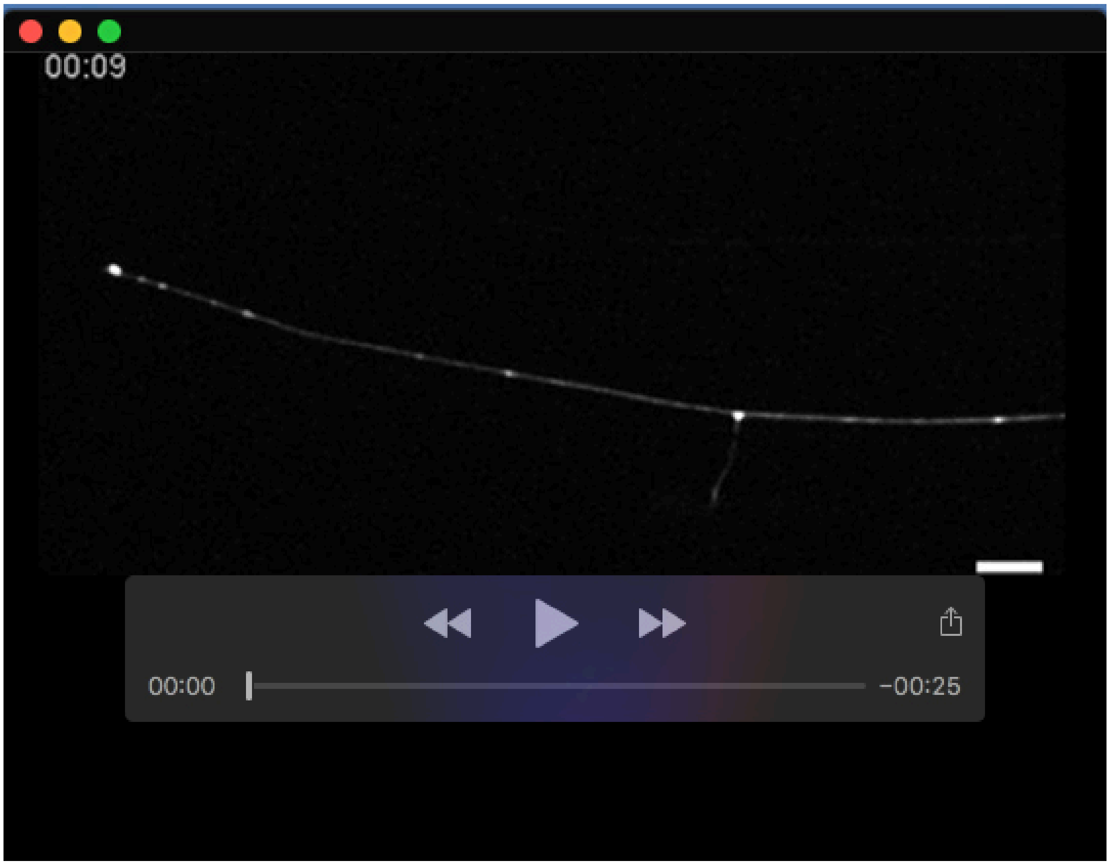

Movie 3. NEKL-3 movement in the distal end of the PLM axon. NEKL-3 is more stationary in the distal region of the PLM axon. NEKL-3::SCARLET puncta were analyzed using the *Pmec-7::nekl-3::scarlet* transgene that expresses NEKL-3::SCARLET in touch receptor neurons. An image was taken at one second intervals for two minutes total with a 40x water objective on a spinning disk confocal microscope.ve. Scale bar represents 10μm.

Development • Supplementary information
